# Protective Effect of Trimetazidine on Potassium Ion Homeostasis in Myocardial Tissue in Mice with Heart Failure

**DOI:** 10.1155/2022/2387860

**Published:** 2022-01-19

**Authors:** Kaijing Yang, Yitao Xue, Miao Yu, Huachen Jiao, Yan Li, Xijin Wei, Wenwen Liu, Yang Sun, Nannan Chen, Linlin Song, Ting Yu, Kaiming Chen, Dadong Guo

**Affiliations:** ^1^Shandong University of Traditional Chinese Medicine, Jinan 250011, China; ^2^Affiliated Hospital of Shandong University of Traditional Chinese Medicine, Jinan 250011, China; ^3^Traditional Chinese Medicine Hospital of Jimo District, Qingdao 266200, China; ^4^Department of Laboratory of Molecular Diagnosis and Regenerative Medicine, Affiliated Hospital of Qingdao University, Qingdao 266000, China; ^5^Shandong Provincial Key Laboratory of Integrated Traditional Chinese and Western Medicine for Prevention and Treatment of Eye Diseases; Shandong Academy of Eye Disease Prevention and Therapy; Affiliated Eye Hospital of Shandong University of Traditional Chinese Medicine, Jinan 250002, China

## Abstract

The occurrence of heart failure (HF) is closely correlated with the disturbance of mitochondrial energy metabolism, and trimetazidine (TMZ) has been regarded as an effective agent in treating HF. Intracellular potassium ion (K^+^) homeostasis, which is modulated by K^+^ channels and transporters, is crucial for maintaining normal myocardial function and can be disrupted by HF. This study is aimed at exploring the protective effect of TMZ on K^+^ homeostasis within myocardial tissue in mice with HF. We observed the pathological changes of myocardial tissue under microscopes and further measured the content of adenosine triphosphate (ATP), the activity of Na^+^-K^+^ ATPase, and the expression of ATP1*α*1 at the mRNA and protein levels. Moreover, we also analyzed the changes in K^+^ flux across the myocardial tissue in mice. As a result, we found that there was a large amount of myocardial fiber lysis and fracture in HF myocardial tissue. Meanwhile, the potassium flux of mice with HF was reduced, and the expression of ATP1*α*1, the activity of Na^+^-K^+^ ATPase, and the supply and delivery of ATP were also decreased. In contrast, TMZ can effectively treat HF by restoring K^+^ homeostasis in the local microenvironment of myocardial tissues.

## 1. Introduction

Heart failure (HF) is defined as a complex clinical syndrome in which ventricular filling or ejection function is damaged due to various organic or functional heart diseases, accompanied by reduced cardiac output. HF is the main cause of death from cardiovascular diseases and seriously threatens human life and health, affecting approximately 23 million patients worldwide [1, 2]. To date, a large amount of evidence has shown that the occurrence of HF is closely correlated with mitochondrial dysfunction [3]. The heart produces myocardial energy through the oxidation of glucose and fatty acids, thereby regulating heart function and efficiency [4]. In HF, the oxidative metabolism of mitochondria is impaired, and the oxidation of mitochondrial fatty acids exceeds the oxidation of mitochondrial glucose, thus causing pyruvate to accumulate and convert into lactic acid, further accelerating cell apoptosis [5, 6]. Trimetazidine (TMZ) is a fatty acid oxidation inhibitor that inhibits 3-ketoacyl CoA thiolase, and it can enhance glucose oxidation and improve cardiac function [7]. Li et al. found that TMZ can convert the utilization of metabolic substrates from fatty acids to glucose to meet energy requirements, thereby reducing the metabolic remodeling of rats with HF [8].

The heart is an organ with high energy demand and needs much energy (that it consumes in the form of adenosine triphosphate which comes from the oxidative metabolism of mitochondria) to maintain its vitality and pump function [9]. Adenosine triphosphate (ATP), in fact, acts as the “molecular currency” for energy transfer in cells, and mitochondria are at the core of ATP production. Na^+^-K^+^ ATPase is a common target of drugs and diseases that is closely related to energy metabolism [10]; moreover, it is also involved in the main efflux mechanism of Na^+^ in cardiomyocytes. ATP1*α*1 belongs to the Na^+^-K^+^ ATPase subfamily and is regarded to be uniformly expressed in all cells [11]. As a muscle membrane pump, Na^+^-K^+^ ATPase maintains a transmembrane Na^+^ gradient, contributing to ion homeostasis [12, 13]. It is known that the cardiac action potential is generated by the coordinated activation and inactivation of ion channels, which conduct depolarization (Na^+^ and Ca^2+^) currents and repolarization (K^+^) currents [14]. In HF models of humans and other animal species, the level of Na^+^ in cardiomyocytes is upregulated, whereas the outward K^+^ current is downregulated [15]. Hegyi et al. found that the reduction of the K^+^ current, enhancement of the Na^+^/Ca^2+^ exchange, and elevation of the subsequent Na^+^ current contribute to extending the duration of the HF action potential [16]. Hypokalemia (serum [K^+^] < 3.5 mm) also exhibits a significant inhibitory effect on Na^+^-K^+^ ATPase [17]. Moreover, studies have also shown that the level of Na^+^-K^+^ ATPase in patients with HF can be reduced by 40% [18]. Wang et al. found that polydatin and vitamin C can increase the activity of Na^+^-K^+^ ATPase in the myocardium of rats with HF induced by doxorubicin (DOX) and increase the content of ATP through antioxidant effects and energy improvement, thus enhancing myocardial function and exerting a protective role [19]. In addition, Mewes et al. showed that ATP concentration increases with incremental increases in intracellular sodium concentration ([Na^+^]) to 150 mM, accompanied by the elevation of Na^+^-K^+^-ATPase transcription activity [20]. Therefore, the occurrence of HF is closely associated with energy metabolism disorders and the regulatory role of Na^+^-K^+^ ATPase.

Clinical experiments have proven that TMZ, an antiangina pectoris drug, has shown cardioprotective effects in many cardiovascular diseases [21]. It can efficiently reduce brain natriuretic peptide (BNP) levels in HF patients [22]. TMZ can also improve the classification of NYHA heart function (NYHA) levels and increase exercise tolerance to enhance HF patients' activity tolerance and improve their quality of life [23]. In the present study, we speculated that TMZ could enhance myocardial cell Na^+^-K^+^ ATPase activity and maintain K^+^ homeostasis to ensure the supply and delivery of ATP in an HF mouse model and increase the expression of ATP1*α*1 to protect the structure and function of mitochondria, thus improving heart energy metabolism to treat HF. In this regard, we explored the molecular mechanisms of TMZ in the treatment of HF. Our investigations will pave the way for TMZ to treat HF in clinical practice.

## 2. Materials and Methods

### 2.1. Experimental Materials

#### 2.1.1. Experimental Agents

TMZ hydrochloride tablets were supplied by Servier Pharmaceutical Co., Ltd. (Tianjin, China), while doxorubicin was produced by Dalian Meilun Biotechnology Co., Ltd. (Dalian, China).

#### 2.1.2. Experimental Animals

The entire experiment was approved by the Experimental Animal Ethics Committee of the Affiliated Hospital of Shandong University of Traditional Chinese Medicine (AWE-2019-01). The study design was aimed at reducing the discomfort and stress of the animals. All rats were fed and maintained in accordance with the “Guiding Opinions on Treating Laboratory animals” issued by the Ministry of Science and Technology of the People's Republic of China (2006). In this study, 60 male C57BL/6 mice (8 weeks old, weighing approximately 20 g) were provided by Beijing Vital River Laboratory Animal Co., Ltd. (Beijing, China). The animal license number was SCXK (Beijing) 2016-0006, and the certificate number was No. 11400700381280.

#### 2.1.3. Animal Models and Groupings

Sixty healthy C57BL/6 mice (8 weeks old, male) were randomly divided into a normal control (NC) group, a HF model (MOD) group, and a TMZ treatment (TMZ) group. All mice were injected with doxorubicin (3 mg/kg/biw) for 6 weeks to induce HF [24], except those in the NC group. BNP and cardiac ultrasound were used to verify whether HF was successfully induced. On day 1 of the second week, the mice in the TMZ group received TMZ (10 mg/kg/d) for 6 continuous weeks by oral gavage to treat HF, while those in the NC and MOD groups received the same volume of sterilized distilled water. Six weeks later, the mice in each group were fasted for 12 h after the last administration and were weighed and anesthetized by intraperitoneal injection of 3% sodium pentobarbital (50 mg/kg). Subsequently, the thoracic cavity was cut, the heart was quickly isolated, and myocardial tissue samples were collected.

### 2.2. Echocardiography

The mice in each group received relevant treatments for 6 weeks. At the indicated time, the mice received an injection of sodium 1% pentobarbital (50 mg/kg) for the aim of anesthetization. Next, the changes in the left ventricular ejection fraction (LVEF) of the mice were detected by an ultrasound B-mode system (M5 Vet, Mindray Biomedical Electronics Co., Ltd., Shenzhen, China).

### 2.3. BNP Levels

Prior to sacrifice, the mice were anesthetized by intraperitoneal injection of 3% pentobarbital (50 mg/kg) until they were in a deep coma and the pain reflex disappeared. Subsequently, 1 ml of blood from the inferior vena was collected and centrifuged at 600 g for 10 min to collect the plasma. A Mouse Brain Natriuretic Peptide Enzyme-linked Immunoassay Kit (Wuhan Huamei Biological Engineering Co., Ltd, Wuhan, China) was used to determine the BNP level to validate whether the model was successfully established.

### 2.4. Histopathological Staining

After different interventions for 6 weeks, mice in each group (*n* = 5) were anesthetized, and the myocardial tissues were extracted, placed in cold 24-well plates (NEST Biotechnology, Wuxi, China), fixed with 4% paraformaldehyde, and then embedded in paraffin. After cutting into 5 *μ*m sections, each section was stained with hematoxylin and eosin (H&E) solution. Finally, the slides were observed under an optical microscope (Eclipse55i; Nikon, Tokyo, Japan) with NIS elements D3.2 software (Nikon, Tokyo, Japan).

### 2.5. Transmission Electron Microscopy

After anesthesia with 3% sodium pentobarbital, the left ventricular myocardium was cut and immediately placed in 4% glutaraldehyde fixative (<2 min). After fixation, dehydration, embedding, and polymerization, ultrathin sections of 60-80 nm were made, and lead citrate staining was performed. Finally, a transmission electron microscope (HITACHI, Japan) was used to observe the mitochondrial ultrastructure of cardiomyocytes.

### 2.6. ATP Levels

In this study, we measured the ATP level in myocardial tissues in each group (*n* = 6). The mice were sacrificed after anesthesia, and the myocardial tissues were isolated. Then, the tissues and 0.9% NaCl (weight (g): volume (ml) = 1 : 4) solution were placed into enzyme-free 5 ml plastic tubes (NEST Biotechnology, Wuxi, China), homogenized for 10 min, and centrifuged at 2500 rpm at 4°C for 10 min to collect the supernatants. The ATP level was measured with an ATP determination kit (Nanjing Jiancheng Institute of Bioengineering, Nanjing, China) in accordance with the manufacturer's instructions. The protein level of the supernatants in each group was measured using a BCA protein concentration measurement kit (Sparkjade Science Co., Ltd., China).

### 2.7. Na^+^-K^+^ ATPase mRNA Levels

Total RNA in each group (*n* = 5) was extracted from the cardiac muscle tissue using an RNA tissue/cell rapid extraction kit (Shandong Sparkjade Science Co., Ltd., China) and quantified with a K5600 spectrophotometer (K5600; Beijing Kaiao Technology Development Co., Ltd., Beijing, China). Then, a HiScriptIIQ RT SuperMix for Q-PCR kit (Vazyme Biotechnology Co., Ltd., Nanjing, China) was used to synthesize complementary DNA (cDNA), and SYBR Q-PCR Master Mix (Vazyme Biotechnology Co., Ltd., Nanjing, China) was used to amplify the target gene using a LightCycler 480 II instrument (Roche Diagnostics, Mannheim, Germany). The target-specific primers are listed in Table 1. The Q-PCR program was set as follows: 1 cycle at 95°C for 30 s, followed by 45 cycles at 95°C for 10 s and 61°C for 30 s. After Q-PCR detection, the 2^-*ΔΔ*Ct^ method was used to calculate the level of the target gene.

### 2.8. Na^+^-K^+^ ATPase Protein Levels

To measure the Na^+^-K^+^ ATPase protein level in myocardial tissue in each group, enzyme-linked immunosorbent assay (ELISA) was carried out. In the present study, mice in each group (*n* = 6) were sacrificed, and then, the myocardial tissue and phosphate-buffered saline (PBS, pH 7.4) solution (weight (g): volume (ml) = 1 : 9) were placed into enzyme-free 5 ml plastic tubes (NEST Biotechnology, Wuxi, China), homogenized for 20 min, and centrifuged at 3000 rpm at 4°C for 20 min. Then, the supernatants were collected. Subsequently, a mouse sodium-potassium ATPase transporter a1 (ATP1*α*1) ELISA kit (Shanghai Jianglai Biotechnology Co., Ltd., Shanghai, China) was used to detect the ATP1*α*1 protein level. At the same time, the protein level of the supernatants in each group was measured using a BCA protein concentration measurement kit (Shandong Sparkjade Science Co., Ltd., China) according to the manufacturer's instructions. The absorbance of the samples was measured at 450 nm using a microplate reader (Elx800, BioTek Instruments, USA). Each experiment was repeated three times.

### 2.9. Na^+^-K^+^ ATPase Activity

After adding cold saline, the isolated myocardial tissues (*n* = 6) were immersed in cold PBS (weight (g): volume (ml) = 1 : 9). After homogenization, the solution was centrifuged at 2500 rpm for 10 min, and then, the supernatants were used to measure the Na^+^-K^+^ ATPase activity using a Na^+^-K^+^ ATPase activity assay kit (Nanjing Jiancheng Institute of Bioengineering, Nanjing, China). The absorbance value of each sample was measured with a 4802S ultraviolet/visible dual-beam spectrophotometer (Unico (Shanghai) Co., Ltd.) at 636 nm, and each experiment was repeated three times.

### 2.10. K^+^ Flux

To study the effect of HF on myocardial tissue K^+^ flux, noninvasive microtesting technology (NMT) (Younger USA LLC, Amherst, MA, USA) was used to measure the K^+^ flux across the myocardial tissues. Prior to the measurement, C57BL/6 mice were anesthetized, and the heart was exposed in a clean ionic solution environment (0.5 mM KCl, 0.1 mM CaCl_2_, 1 mM NaCl, 0.1 mM KH_2_PO_4_, 0.1 mM NaHCO_3_, 0.1 mM Na_2_HPO_4_, 5.6 mM glucose, and pH 7.2). First, a tissue sample microsensor (*Φ*4.5 ± 0.5 *μ*m, XY-CGQ -01, Younger, USA) was injected into a column of electrolyte solution (100 mM KCl) with a length of 1 cm at a distance of 1 cm from the tip. Then, a syringe was used to exert pressure on the flow sensor to gradually fill the tip of the flow sensor with electrolyte. A carrier glass electrode through a liquid ion exchanger (Liquid Ion-eXchange holder, LIX holder, XY-LIX-01, Younger, USA) was injected into a liquid ion exchanger (Liquid Ion-eXchange, LIX, XY-SJ-K, Younger USA) with a length of 180 *μ*m into the tip, forming a K^+^ flow sensor. After inserting the chlorinated silver wire from the back end of the K^+^ flow sensor, the tip of the silver wire was immersed in the electrolyte and kept a certain distance from the tip of the K^+^ flow sensor. After the sample testing was calibrated in the ion solution (1 mM KCl/0.1 mM KCl, 0.1 mM CaCl_2_, 1 mM NaCl, 0.1 mM KH_2_PO_4_, 0.1 mM NaHCO_3_, 0.1 mM Na_2_HPO_4_, 5.6 mM glucose, and pH 7.2), the K^+^ flux determination was initiated. Fick's diffusion law formula was used to calculate the K^+^ flux: *J* (picomole · cm^−2·^s^−1^) = −*D*_0_ (*dc*/*dx*), where *D*_0_ is the diffusion coefficient, *dc* represents the K^+^ concentration difference between the two test points, and *dx* represents the distance between two test points (5-35 *μ*m).

### 2.11. Statistical Analysis

Each experiment was repeated three times, and data were presented as the mean ± SD (standard deviation). The statistical analysis was performed using statistical software (SPSS for Windows, version 17.0, Chicago, USA) with one-way ANOVA followed by Tukey's multiple comparison test. *P* < 0.05 was considered to be statistically significant.

## 3. Results

### 3.1. Effect of TMZ on Systolic Left Ventricular Function

**Figure 1:**
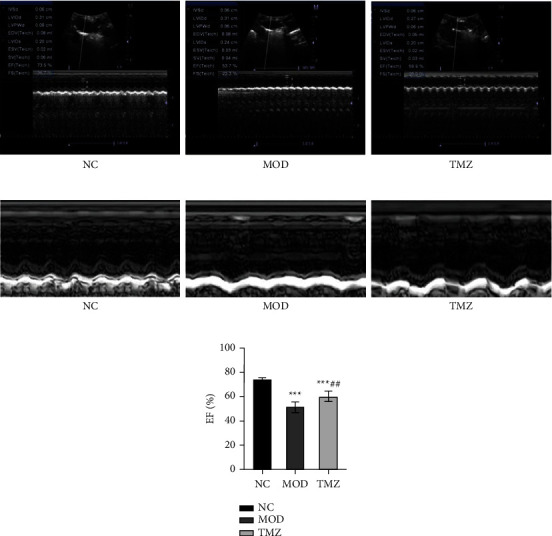
Change in cardiac function in the NC, MOD, and TMZ groups. After modeling, decreased cardiac function was found in the MOD group. However, after TMZ treatment, cardiac function was restored (a, b). (c) A graph representing ejection fraction. ^∗∗∗^*P* < 0.001 vs. NC group; ^##^*P* < 0.01 vs. MOD group.

After the relevant treatments for 6 weeks, the ejection fraction (EF) of the mice in the MOD group was significantly reduced compared with that of the NC group (51.36 ± 3% vs. 73.59 ± 2%, ^∗∗∗^*P* < 0.001, Figure 1). However, after TMZ treatment, we noted that the EF of the mice in the TMZ group was apparently restored compared with that of the MOD group (60.34 ± 4% vs. 51.36 ± 3%, ^##^*P* < 0.01, Figure 1), indicating the good therapeutic effect of TMZ on HF.

### 3.2. BNP Level

The comparison of BNP levels in serum between the MOD and NC groups showed that the BNP level in the MOD group was significantly higher than that in the NC group, and the difference was statistically significant (Table 2, ^∗∗∗^*P* < 0.001), indicating that the HF model of mice was successfully established. We also noted that after TMZ treatment, the BNP level decreased significantly compared with that of the MOD group (Table 2, ^##^*P* < 0.01).

### 3.3. Pathological Change in Myocardial Tissues

**Figure 2:**
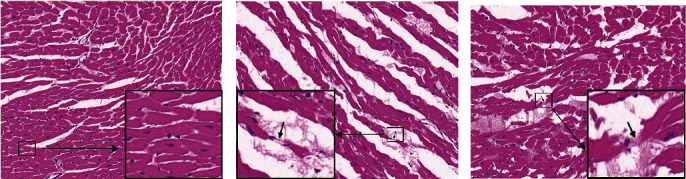
Histopathological characterization of the myocardial tissues of C57BL/6 mice in each group. After different treatments, the mice in each group were sacrificed, and the myocardial tissues were isolated and sectioned. After staining with hematoxylin and eosin (H&E) solution, the sections were observed under a microscope. Magnification: 200x. (a) Normal control group, (b) model group, and (c) TMZ group (10 mg/kg/d).

Histopathological examination showed that the myocardial tissues of the C57BL/6 mice in the NC group were in good alignment, the myocardial fiber morphology and structure were normal, the boundaries were clear, the arrangement was regular, and there was no obvious abnormality in the interstitium (Figure 2(a)). However, a large amount of myocardial fiber lysis and fracture (black arrow), a small amount of myocardial fiber vacuole degeneration, circular vacuoles of various sizes, and eosinophilic substances were observed in the tissues from the MOD group (Figure 2(b)). Meanwhile, a small amount of myocardial fibrillation (black arrow) and eosinophilic substance exudation were observed locally in the myocardial tissue of the mice in the TMZ group (Figure 2(c)).

### 3.4. Change in Ultrastructure of Myocardial Mitochondria

**Figure 3:**
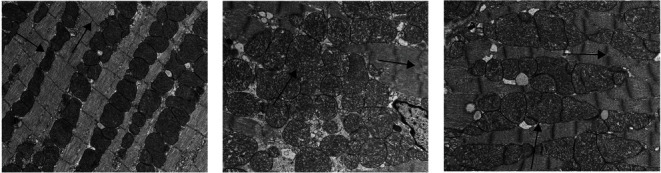
Mitochondrial ultrastructure of cardiomyocytes: (a) NC group; (b) MOD group; (c) TMZ group.

As shown in Figure 3, the mitochondria of the left ventricular myocardium in the NC group were well proportioned, well balanced in size, and regularly arranged. The mitochondrial cristae were clear and complete, the membranes were intact, sarcomeres were arranged neatly, horizontal stripes were clear, bright and dark bands were clearly visible (Figure 3(a), arrows), and the structure of each part was complete. In contrast, mitochondria in the MOD group varied in size, with more than 1 *μ*m in transverse diameter, and there was also mitochondrial swelling and hypertrophy, displacement and aggregation with loss of mitochondrial membrane, mitochondrial crista dissolution and breakage, and disordered or missing muscle fiber arrangement (Figure 3(b), arrows). After TMZ treatment, there were myocardial mitochondrial edema vacuole-like changes, some mitochondrial cristae were blurred or even broken, and the arrangement of muscle fibers was disordered, but to a lighter extent than in the MOD group (Figure 3(c), arrows).

### 3.5. Change of ATP Level in Myocardial Tissues

**Figure 4:**
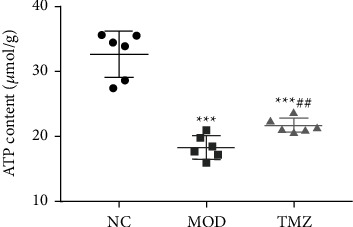
Determination of ATP contents in myocardial tissues in the NC, MOD, and TMZ groups. The results showed that the ATP content of MOD mice was lower than that of the NC group. However, after TMZ treatment, the ATP content in the myocardial tissues was higher than that in the MOD group. Compared to the NC group, ^∗∗∗^*P* < 0.001; compared to the MOD group, ^##^*P* < 0.01. *n* = 6.

To study the changes in ATP content in myocardial tissues after different treatments, we measured the ATP level in myocardial tissues in the NC, MOD, and TMZ groups. As shown in Figure 4, the ATP content in the MOD group was significantly reduced compared with that in the NC group (^∗∗∗^*P* < 0.001). At the same time, it was also found that the ATP content in myocardial tissues in the TMZ group was apparently higher than that in the MOD group (^##^*P* < 0.01, Figure 4). Nevertheless, the ATP content in the TMZ group was lower than that in the NC group (^∗∗∗^*P* < 0.001, Figure 4).

### 3.6. Na^+^-K^+^ ATPase Expression

**Figure 5:**
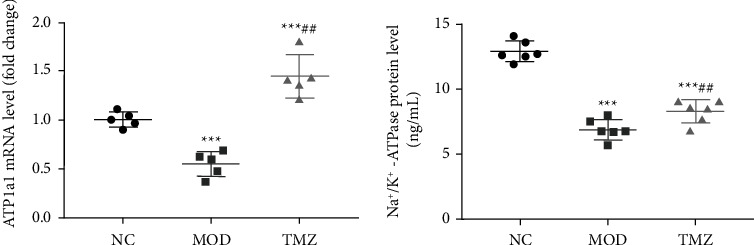
Determination of the expression of ATP1*α*1 at the mRNA and protein levels in myocardial tissues in C57BL/6 mice after different treatments. After relevant treatments, ATP1*α*1 mRNA and protein levels were measured by Q-PCR and ELISA, respectively. The results show that the expression of ATP1*α*1 in the myocardium in the MOD group was significantly lower than that in the NC group. After TMZ treatment, the Na^+^-K^+^ ATPase protein level was significantly elevated compared to that in the MOD group. Compared to the NC group, ^∗∗^*P* < 0.01 and ^∗∗∗^*P* < 0.001; compared to the MOD group, ^#^*P* < 0.05 and ^###^*P* < 0.001. *n* = 5 for the determination of ATP1*α*1 mRNA levels, *n* = 6 for the determination of Na^+^-K^+^ ATPase protein levels.

To explore the effect of different treatments on the expression of Na^+^-K^+^ ATPase in the myocardial tissue of C57BL/6 mice, we measured Na^+^-K^+^ ATPase expression at the mRNA and protein levels in the myocardial tissues of the NC, MOD, and TMZ groups. The results showed that the Na^+^-K^+^ ATPase mRNA level in myocardial tissues in the MOD group was significantly lower than that in the NC group (^∗∗∗^*P* < 0.001, Figure 5(a)). Similarly, the Na^+^-K^+^ ATPase protein level in the myocardial tissues in the MOD group was also significantly lower than that in the NC group (^∗∗∗^*P* < 0.001, Figure 5(b)). In contrast, TMZ treatment markedly elevated Na^+^-K^+^ ATPase expression at the mRNA and protein levels compared to the MOD group (^###^*P* < 0.001 and ^#^*P* < 0.05, Figures 5(a) and 5(b)).

### 3.7. Changes in Na^+^-K^+^ ATPase Activity

**Figure 6:**
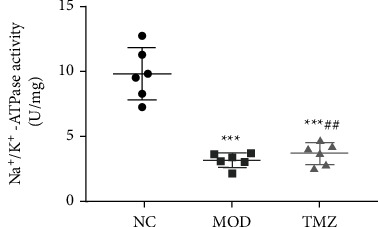
Determination of Na^+^-K^+^ ATPase activities in the myocardial tissues of C57BL/6 mice after different treatments. An HF mouse model was induced by doxorubicin administration, and the mice received different treatments for 6 weeks. At the indicated time, the mice were sacrificed after anesthesia, and myocardial tissues were isolated, ground under liquid nitrogen, and centrifuged at 2500 rpm for 10 min. Then, the supernatants were collected to measure Na^+^-K^+^ ATPase activity. Compared to the NC group, ^∗∗∗^*P* < 0.001; compared to the MOD group, ^#^*P* < 0.05. *n* = 6.

As shown in Figure 6, the results show that compared with that of NC mice, the Na^+^-K^+^ ATPase activity in the MOD group was significantly reduced (^∗∗∗^*P* < 0.001, Figure 6). However, TMZ treatment apparently elevated Na^+^-K^+^ ATPase activity compared with that of the MOD group (^#^*P* < 0.05, Figure 6), indicating that TMZ treatment can efficiently enhance the activity of Na^+^-K^+^ ATPase.

### 3.8. Measurements of K^+^ Flux

**Figure 7:**
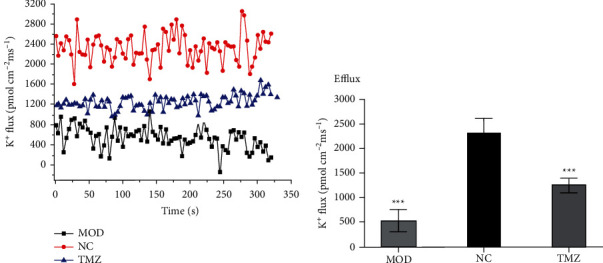
Measurement of K^+^ flux across the myocardial tissue in C57BL/6 mice in the NC, MOD, and TMZ groups. After relevant treatments, C57BL/6 mice were first anesthetized, the myocardial tissue was exposed, and then NMT was used to measure the real-time K^+^ flux across the myocardial tissue (a). Histogram analysis shows the trend of K^+^ flux measured by the NMT technique in C57BL/6 mice in the TMZ group (b). ^∗∗∗^*P* < 0.001 vs. NC group.

To explore the influence of various treatments on K^+^ flux in myocardiocytes, we determined the real-time K^+^ flux using NMT. As shown in Figure 7, K^+^ flux across the myocardial tissue from normal mice shows an outflow trend and is at a high level. In contrast, the K^+^ flux in the MOD group was near the static state, indicating seriously damaged myocardial function. However, after TMZ treatment, we noted that the K^+^ flux was apparently restored, indicating enhanced myocardial function.

## 4. Discussion

As a rapidly growing public health issue and a clinically complex chronic disease, HF seriously affects the quality of life of patients [25, 26]. In recent years, the regulation of cardiac energy metabolism by reducing fatty acid oxidation and/or increasing glucose oxidation has been a new way to treat HF. TMZ is a metabolic regulator that is widely used in the treatment of stable angina pectoris, and there is a relatively large amount of evidence demonstrating that it can be used to treat HF [27]. Studies have shown that TMZ exerts its anti-ischemic effect by regulating the metabolism of myocardial tissue mitochondria without changing hemodynamic function and has no effect on coronary flow, contractility, blood pressure, or heart rate [28]. Current research shows that TMZ can inhibit fatty acid oxidation and increase glucose oxidation, thereby producing ATP and consuming less oxygen [29]. In addition, Chen et al. showed that TMZ can reduce LPS-induced cardiomyocyte apoptosis and cardiac dysfunction by promoting the recruitment of neutrophils to cardiac tissue through CXCR2 [30]. Moreover, TMZ can also protect against Ca^2+^ overload and Ca^2+^ release in arrhythmia, thus maintaining sufficient contractility of HF myocardium [31], improving antioxidant levels, decreasing oxidative stress, and reducing plasma BNP levels [32]. To investigate the protective effect of TMZ on the myocardium in HF mice and the underlying mechanism, we established a doxorubicin-induced HF mouse model. Our results show that TMZ treatment effectively reduced the BNP level in the serum of HF mice, which is released from ventricular myocytes due to myocardial stress, such as myocardial infarction and HF. Histopathological examination showed that normal mouse myocardial tissues were arranged neatly and structurally intact, while the myocardium in HF mice was disorderly arranged and severely damaged, and there were multiple myocardial dissolutions and myofilament ruptures. These results are consistent with the study by Al-Harthi et al. [33].

The high energy demand of the heart is mainly met by mitochondrial oxidation of fatty acids and glucose [34]. Mitochondrial oxidative phosphorylation uses fatty acids, glucose, lactic acid, and ketone bodies as the main substrates to produce ATP [35]. However, the glucose oxidation metabolism of heart mitochondria will be reduced in HF, which will lead to insufficient energy of myocardial tissue. Myocardial energy metabolism disorder is one of the important causes of HF [36, 37], and mitochondrial dysfunction is the driving force involved in the imbalance of myocardial energy supply and demand [38], which ultimately causes a lack of ATP in myocardial tissue and impaired myocardial contractility [39]. The free energy produced by ATP hydrolysis (*Δ*G~ATP) is an important indicator of myocardial energy. If myocardial cells are in a state of ischemia or hypoxia, *Δ*G~ATP will decrease, and cardiac contractility will be weakened, eventually leading to HF [40]. Under normal physiological conditions, the oxidative phosphorylation of mitochondria in cells can produce adequate ATP [41], and its intracellular function is mainly to store and transmit chemical energy so that various life activities of the cell can obtain sufficient energy support. It is well known that the heart is an organ with high oxygen consumption, and myocardial tissue needs ATP hydrolysis to generate a large amount of energy to ensure the normal operation of its own electromechanical activities. Considering that Na^+^-K^+^ ATPase requires energy from ATP hydrolysis to maintain normal physiological functions, it is essential for the electrical excitability of cells to maintain the balance of cell membrane potential. Thus, energy metabolism disorders will seriously affect the activity of Na^+^-K^+^ ATPase. When the homeostasis of ions within the cell is destroyed, cell function will be impaired, and eventually, HF develops. Panagia et al. have shown that the inhibition of acute reversal of mitochondrial ATP production can improve systolic dysfunction in metabolic HF [42]. In our study, we noted that the myocardial tissues in mice with HF exhibited decreased ATP production, indicating that HF could damage the capacity of ATP production in myocardial tissues and thus lead to dysfunction of ATP metabolism. Treatment with TMZ can enhance ATP synthesis, prevent transmission obstacles, increase ATP generation, improve myocardial energy metabolism to a certain extent, and finally rehabilitate heart function. TMZ acts as an inhibitor of free fatty acid oxidation, selectively inhibits long-chain 3-ketoacyl-CoA thiolase, and directly stimulates pyruvate dehydrogenase, which converts cardiac energy metabolism from fatty acid oxidation to glucose oxidation to preserve the level of ATP necessary within the cells of the myocardium [43].

Moreover, we also noted that TMZ can increase the expression of ATP1*α*1 and stimulate the activity of Na^+^-K^+^ ATPase to a certain extent compared with HF mice. Na^+^-K^+^ ATPase is a P-type ion translocating adenosine triphosphatase that can couple ATP hydrolysis with the transport of three Na^+^ ions out and two K^+^ ions into cells to maintain the penetration and ion balance between the internal and external environments of the cells [44–46]. The enzyme contains two main subunits (*α* and *β*), of which *α* subunits exist in the form of four subtypes (*α*1-*α*4), encoded by four different genes ATP1*α*1-4 [47, 48]. Dostanic et al. found that ATP1*α*1 isoforms can regulate cardiac contractility. At high concentrations of ouabain, the activity of ATP1*α*1 subtypes is significantly reduced, HF occurs, and ATP1*α*1 subtypes account for most of the mouse heart [49]. Therefore, inhibition of Na^+^-K^+^ ATPase activity can lead to HF. The activity of Na^+^-K^+^ ATPase in the myocardial tissue of the MOD group was reduced, and the expression of ATP1*α*1 was inhibited, which was consistent with our experimental results. Correll et al. found that the removal of Na^+^ from the cytoplasm of cardiomyocytes is mainly accomplished by Na^+^-K^+^ ATPase-*α*1, which accounts for 88% of cardiac Na^+^-K^+^ ATPase activity [50]. Moreover, a large amount of evidence shows that Na^+^-K^+^ ATPase can regulate cardiac contractility. As a ubiquitous subtype, the *α*1 subtype is dominant in cardiomyocytes with pumping and signaling functions and usually changes in hypertrophic and failing hearts [51, 52]. Furthermore, Na^+^-K^+^ ATPase is also the only known receptor for cardiotonic steroids (such as ouabain) [53]. It has been reported that digitalis can be used to treat HF because it can inhibit the pumping function of Na^+^-K^+^ ATPase and stimulate the signal conduction function [54]. Na^+^-K^+^ ATPase is also an important mediator of vasculature tension and contractility, and its abnormal expression can induce myocardial cell death and cardiac dysfunction, possibly leading to myocardial expansion and HF [55].

Hisatome et al. found that TMZ has a slight inhibitory effect on Na^+^-K^+^ ATPase [56]. In contrast, this study found that the Na^+^-K^+^ ATPase activity of mice treated with TMZ was higher than that of the HF group. We believe that the difference in the results is due to the more severely inhibited Na^+^-K^+^ ATPase activity in mice with HF. Moreover, growing evidence shows that Na^+^-K^+^ ATPase is closely related to cardiac electrophysiology and myocardial contractility. In fact, changes in Na^+^-K^+^ ATPase activity can affect skeletal muscle absorption or the release of K^+^ [57]. During muscle fiber excitement, the action potential is associated with a significant increase in Na^+^ influx and K^+^ efflux [58]. Under normal physiological conditions, the action potential consists of two stages: (1) depolarization caused by the increase in Na^+^ influx and (2) repolarization caused by the increase in K^+^ efflux through the voltage-dependent potassium channel (Kv) [59]. Every normal heartbeat is generated by an action potential (AP) wave, which travels from the atrium to the ventricle, causing the heart to contract and eject blood [60]. To date, a large number of studies have shown that the current of cardiac repolarization will produce important changes when HF occurs, and the downregulation of multiple K^+^ channel currents causes delays in repolarization and prolongs the duration of action potentials [61–63]. In this study, we found that the K^+^ flux rate in HF mouse cardiomyocytes was much lower than that in normal mouse cardiomyocytes. We speculate that K^+^ homeostasis is crucial for maintaining normal myocardial function. The status of K^+^ homeostasis in myocardial tissue in HF mice was destroyed, and TMZ treatment efficiently contributed to restoring the balance of K^+^ flux to maintain the action potential of the heart, thereby improving cardiovascular function.

## 5. Conclusion

In summary, our results indicate that TMZ can efficiently increase the expression of ATP1*α*1 in myocardial tissues in mice with HF, enhance the activity and production of Na^+^-K^+^ ATPase, elevate the content of ATP, and efficiently restore and maintain the balance of K^+^ homeostasis within myocardial cells, thereby improving the energy metabolism of the heart and rehabilitating the balance of the myocardial microenvironment. Our study provides a new theoretical basis for the clinical application of TMZ in the treatment of HF.

## Figures and Tables

**Table 1:** ATP1*α*1 primer sequences for quantitative PCR.

Gene	Primer sequences
ATP1*α*1	F: 5′-GAGGCAGCCCAGAAACCCCAAAAC-3′
ATP1*α*1	R: 5′-TCGGCCCACTGCACTACCACAATA-3′
GAPDH	F: 5′-ACGGCAAATTCAACGGCACAGTCA-3′
GAPDH	R: 5′-CGGCAGAAGGGGCGGAGATG-3′

**Table 2:** Determination of BNP level (*n* = 8).

Groups	BNP level (pg/ml)
NC	158.673 ± 65.867
MOD	485.742 ± 124.613^∗∗∗^
TMZ	343.39 ± 55.393^∗∗^^##^

^∗∗^ *P* < 0.01 and ^∗∗∗^*P* < 0.001 vs. NC group. ^##^*P* < 0.01 vs. MOD group.

## Data Availability

The data sets used and/or analyzed in the current research can be obtained from the corresponding authors according to reasonable requirements.
